# Improving health worker performance: an ongoing challenge for meeting the sustainable development goals

**DOI:** 10.1136/bmj.k2813

**Published:** 2018-07-30

**Authors:** Alexander K Rowe, Guilhem Labadie, Debra Jackson, Claudia Vivas-Torrealba, Jonathon Simon

**Affiliations:** 1 Malaria Branch, Division of Parasitic Diseases and Malaria, Center for Global Health, US Centers for Disease Control and Prevention, Atlanta, Georgia, USA; 2 Department of Maternal, Newborn, Child, and Adolescent Health, World Health Organization, Geneva, Switzerland; 3 Health Section, Programme Division, United Nations Children’s Fund, New York, USA; Correspondence to: A K Rowe axr9@cdc.gov

## Abstract

Improving health worker performance is crucial to accelerating progress in helping children to survive and thrive, say **Alexander K Rowe and colleagues**

**Key messages**

- Although reaching the sustainable development goal for health requires access to quality essential healthcare services (universal health coverage), hundreds of studies have shown inadequate health worker performance in low and middle income countries
- For two decades, the WHO-Unicef Integrated Management of Childhood Illness (IMCI) strategy has been an important initiative for improving healthcare quality for children by strengthening health worker skills, health systems, and family practices
- IMCI’s focus on training health workers has been less effective than originally hoped: after IMCI training, one third of ill children were still not receiving appropriate treatments
- Compared with training alone, larger increases in healthcare quality might result from combining training with other components, such as supervision or group problem solving, or implementing certain multifaceted strategies
- The key to improving healthcare quality is a multilevel, systems oriented approach that monitors, adapts, and innovates, plus a generous dose of persistence and patience

The sustainable development goal for health (SDG 3) and the targets established in the Global Strategy for Women’s, Children’s, and Adolescents’ Health reflect the priority of increasing efforts to improve child health.^1^ ^2^ National governments have committed to ambitious goals of reducing under 5 mortality to 25 or fewer per 1000 live births and newborn mortality to 12 or fewer per 1000 by 2030 in all countries (box, target 3.2). There is much to do: progress on improving child survival must be accelerated in 52 countries to meet these targets.^3^ Achieving SDG targets in only 12 years requires sustained multisectoral efforts, including reducing poverty, strengthening health systems and infrastructure, expanding education (particularly for girls and women), resolving civil conflicts, and improving governance.^2^ ^4^

Within the health sector, to reach SDG 3, countries must achieve universal health coverage (UHC) (box, target 3.8).^2^ UHC requires access to, and use of, quality essential healthcare services, which need sufficient numbers of health workers who adhere to evidence based standards of care. There is, however, a critical shortage of health workers (global estimates show a shortage of 17.4 million in 2013 and predict a shortage of 14.5 million in 2030) 5; and hundreds of studies have shown inadequate health worker performance in low and middle income countries (LMICs), where the majority of child deaths occur.^6^ ^7^ ^8^ ^9^

While the shortage of health workers deserves attention and action, the way forward has been well described in publications by the World Health Organization, including a global strategy,^10^ a code of practice on international recruitment,^11^ a high level commission that developed 10 key recommendations,^12^ and the SDG 3 target (box, target 3c). Therefore, the remainder of this article focuses on improving health worker performance.

**Targets for sustainable development goal 3: ensure healthy lives and promote wellbeing for all at all ages**

3.1 By 2030, reduce the global maternal mortality ratio to less than 70 per 100 000 live births
3.2 By 2030, end preventable deaths of newborns and children under 5 years of age, with all countries aiming to reduce neonatal mortality to at least as low as 12 per 1000 live births and under 5 mortality to at least as low as 25 per 1000 live births
3.3 By 2030, end the epidemics of AIDS, tuberculosis, malaria, and neglected tropical diseases, and combat hepatitis, waterborne diseases, and other communicable diseases
3.4 By 2030, reduce by one third premature mortality from non-communicable diseases through prevention and treatment and promote mental health and wellbeing
3.5 Strengthen the prevention and treatment of substance abuse, including narcotic drug abuse and harmful use of alcohol
3.6 By 2020, halve the number of global deaths and injuries from road traffic accidents
3.7 By 2030, ensure universal access to sexual and reproductive healthcare services, including for family planning, information and education, and the integration of reproductive health into national strategies and programmes
3.8 Achieve universal health coverage, including financial risk protection, access to quality essential healthcare services, and access to safe, effective, quality, and affordable essential drugs and vaccines for all
3.9 By 2030, substantially reduce the number of deaths and illnesses from hazardous chemicals and air, water, and soil pollution and contamination
3a Strengthen the implementation of WHO’s Framework Convention on Tobacco Control in all countries, as appropriate
3b Support the research and development of vaccines and drugs for the communicable and non-communicable diseases that primarily affect developing countries; provide access to affordable essential drugs and vaccines, in accordance with the Doha Declaration on the TRIPS Agreement and Public Health, which affirms the right of developing countries to use to the full the provisions in the Agreement on Trade Related Aspects of Intellectual Property Rights regarding flexibilities to protect public health; and, in particular, provide access to drugs for all
3c Substantially increase health financing and the recruitment, development, training, and retention of the health workforce in developing countries, especially in least developed countries and small island developing states
3d Strengthen the capacity of all countries, in particular developing countries, for early warning, risk reduction, and management of national and global health risks

Health worker performance encompasses availability, clinical competence, responsiveness (providing patient centred care), and productivity (or efficiency).^13^ Although all these elements are important, at the micro level it is the adherence to evidence based standards of care that saves lives, and thus is our emphasis in this article.

The link between adherence to evidence based guidelines and better health is supported by two types of evidence. Firstly, there are the many clinical trials on which the guidelines are based.^14^ Secondly, one can examine correlations between improved adherence (for example, correct application of active management of the third stage of labour) and patient outcomes (such as postpartum haemorrhage rate) from field studies, as illustrated by results from a systematic review on improving health worker performance in LMICs.^15^ Among 10 intervention studies on maternal and child health from eight countries that measured both health worker practice outcomes and patient health outcomes, improvements in both outcome types were highly correlated (Pearson’s correlation=0.87, P=0.001) (fig 1).^16^ ^17^ ^18^ ^19^ ^20^ ^21^ ^22^

**Fig 1:**
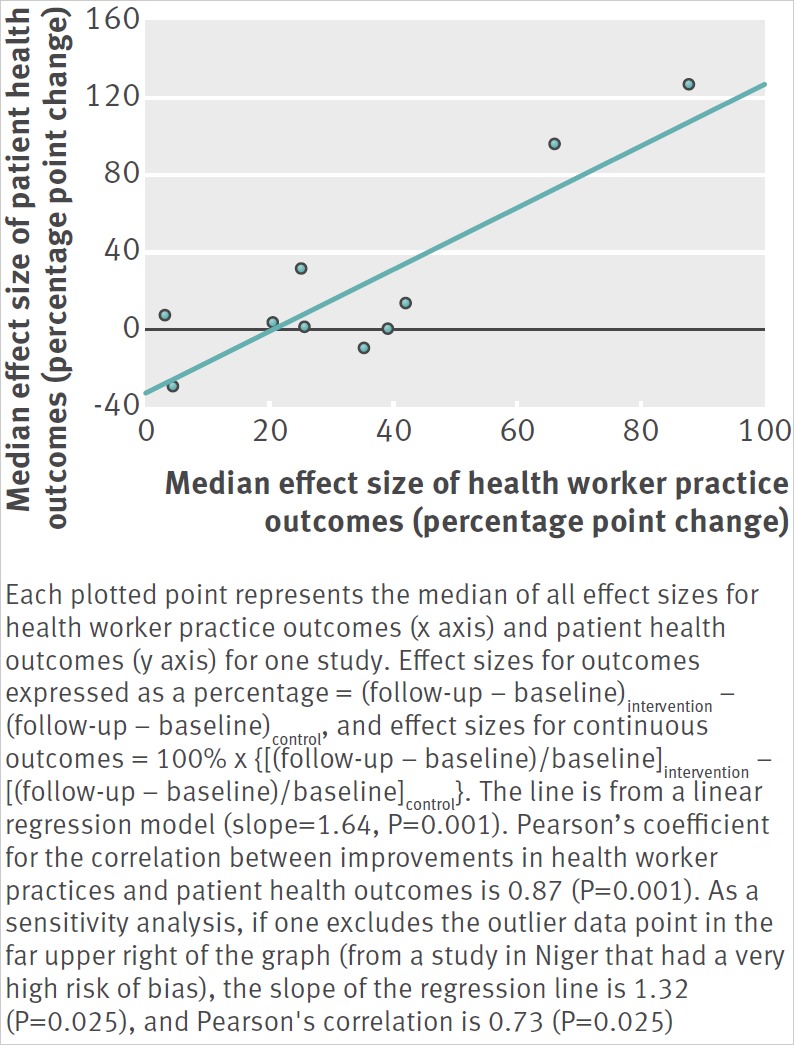
The correlation between improvements in health worker practices and patient health outcomes from 10 studies on maternal and child health in Bangladesh,^16^ Guatemala,^17^ India,^18^ Nicaragua,^19^ Niger,^20^ Pakistan,^21^ Russia,^20^ and Sri Lanka^22^

Having a better understanding of past efforts at improving health worker performance was a key area of inquiry in the recent Child Health Strategic Review conducted by Unicef and WHO. This article presents a history of those efforts, describes the effectiveness of strategies to improve health worker performance, and concludes with recommendations on priority actions for further improvements in this area.

## Historical approaches

The Integrated Management of Childhood Illness (IMCI) strategy, and its associated clinical algorithm, has been an important initiative for two decades.^1^ Although later renamed Integrated Management of Neonatal and Childhood Illness, for simplicity and consistency we will use IMCI throughout the paper, though we cover both time periods.

IMCI has three components: improving health worker skills; improving health systems; and improving family and community practices.^23^

Improving health worker skills has primarily involved adapting case management guidelines, training health workers, and maintaining health worker performance. The core approach was an 11 day training course to teach first level health workers to manage sick children up to 5 years old. The curriculum required at least 30% of the training time in clinical sessions. The importance of supportive supervision after training was foreseen, and a follow-up visit within one month of training was part of the training process.^24^ WHO originally recommended delaying the introduction of IMCI into pre-service training until experience in its use was gained.

Many countries shortened the 11 day course to reduce costs and the time health workers were away from their facilities during training.^25^ A systematic review suggested that the 11 day course was somewhat more effective than shortened training, although the magnitude of the difference was unclear.^26^ The review recommended that countries should consider implementing strategies to support health workers after IMCI training, regardless of training duration (for example, a package of strengthened supervision, job aids, and non-financial incentives).^27^

## Accomplishments and limitations

The Child Health Strategic Review included a survey (the IMCI Global Survey^28^) of country experiences in child health, including efforts to improve health worker performance with IMCI. Nearly all (86 of 94) countries that responded to the survey reported implementing IMCI as a national child health strategy, and 77% of countries reported that at least three quarters of their districts were implementing IMCI. The survey confirmed that many countries (42%) used an abridged course.

The survey showed that key barriers to scaling up IMCI included inadequate budgets for training, human resource problems (staff turnover, retention, and motivation), weak mentorship and supervisory systems, and insufficient facility readiness (drug procurement and supply chain management). More generally, these barriers illustrate the causes of poor health worker performance.

The survey also found that about three quarters of countries implemented IMCI pre-service training, including training for community health workers in newborn care. However, pre-service education was less likely to be scaled up in countries with higher child mortality rates, and IMCI was often not optimally incorporated into curriculums. Ethiopia and Nigeria, for instance, added IMCI as a block at the end of paediatric training. By contrast, the WHO Eastern Mediterranean region developed a standard stepwise approach for IMCI pre-service education, monitored by well defined indicators and supported with tools for each step to ensure institutionalisation and quality.^29^

Overall, IMCI training was far less extensive or effective than originally hoped. After IMCI in-service training (of any duration), 34% of ill children needing oral antimicrobials or rehydration were not getting these treatments according to IMCI guidelines.^26^ To tackle both the coverage and quality problems, an IMCI computerised adaptation and training tool (ICATT) became available in 2006, and a distance learning course (dIMCI) was created in 2014.^30^ These adaptations were designed to lower costs and bring IMCI into the digital education arena. The IMCI global survey found that at least 14% of countries used ICATT and 2% used dIMCI.^28^

## Strategies to improve health worker performance

Over the past five decades, a large body of evidence has emerged on the effectiveness of strategies to improve health worker performance in LMICs. Several systematic efforts to gather and synthesise this evidence either have been recently completed or soon will be available: the Health Care Provider Performance Review (HCPPR)^15^; the Lancet Global Health Commission on High Quality Health Systems in the SDG Era^31^; the Network for Improving Quality of Care for Maternal, Newborn, and Child Health^32^; and several others.^33^ ^34^ ^35^ ^36^

Results from the HCPPR, which includes studies of patients of all ages, suggest that some commonly used strategies tended to have modest effects (for example, training or supervision only showed a median improvement of 7 to 12 percentage points) or almost no effect (such as when only printed information was provided to health workers). For context, the mean performance level at baseline for these studies was 38%. By contrast, training plus other components seemed more effective. For example, the improvement from training plus supervision ranged from 10 to 26 percentage points, and the median effect of training plus group problem solving was 61 percentage points (although this result had limited generalisability). Some complex, multifaceted strategies had large effect sizes (for example, the combination of training, supervision, printed information for health workers and patients, providing commodities, social marketing, accreditation, and establishing a referral network, with effects ranging from 30 to 38 percentage points),^37^ however multifaceted strategies were not consistently more effective than simpler ones.

Although the high coverage of mobile phone services in LMICs (an estimated 93% among all Africans)^38^ creates an opportunity for mobile health strategies, the impact of such strategies needs better characterisation. Few well done trials have been conducted, and results are inconsistent. For example, the effect of sending text messages to health workers’ phones to remind them of key aspects of clinical guidelines ranged from almost no effect^39^ to a 24 percentage point improvement.^40^

For all strategies tested by multiple studies, effects ranged substantially (for example, among 36 studies of low intensity training, the interquartile range of effects was 3 to 23 percentage points; minimum and maximum were -16 and 55 percentage points). This wide variability shows the difficulty in predicting how effective a strategy will be in a given context. Even after implementing strategies that studies show are relatively effective, performance gaps are likely to exist, which underscores the importance of monitoring performance and suggests the need to layer multiple strategies over time.

## Conclusions and recommendations

To attain SDG3, countries—with support from development partners, the private sector and others—need to pursue a multipronged approach that achieves UHC by increasing the access and quality of health services, identifies new health technologies, and makes advances in non-health sectors, such as general economic growth, education, and water, sanitation, and hygiene. Overwhelming evidence of the inadequate quality of care delivered to many patients in LMICs justifies increased attention to improving health worker performance. Actions to improve performance should benefit all age groups, not only children.

At the local level, a practical approach, adapted from quality improvement methods, is for programmes to implement an initial strategy based on research evidence and understanding the local context; monitor performance and provide feedback; identify remaining quality and coverage gaps (and not be discouraged by them); modify the strategy or add a new one; and continue to iteratively monitor and adjust the strategy. Performance could be monitored by health workers themselves (for example, by graphing results extracted from patient registers), supervisors, or district managers, as well as through continuous surveys.^41^ This general improvement approach can begin in a small area, and successful strategies can be scaled-up.

Nationally, it is crucial to support local improvement efforts by strengthening health systems by assuring the supply of essential commodities and equipment; improving the capacity for monitoring quality (through enhanced health management information systems, for example) and using the data for decision making; prioritising supportive supervision; and fostering a stronger culture that emphasises quality. The basic needs of health workers should be provided (such as a liveable wage or housing for workers in rural areas) to maintain morale and encourage providers to work long term in poor areas with the greatest health needs. Investments to increase the number of health workers would also be critical in countries with workforce shortages.

At the global level, development partners can intensify advocacy for improved health worker performance and provide substantially more funding and technical assistance; fund new research; support efforts to review new evidence and revise guidance on both clinical practice and quality improvement practice; and facilitate learning among countries and programmes. Development partners can serve as a mechanism for accountability, so the global community can see the extent to which quality is actually improving over time and place. Additionally, support is needed for initiatives such as the WHO Global Code of Practice on the International Recruitment of Health Personnel for managing the large number of health workers that migrate from LMICs to high income countries.^11^

The 12 years available before the world evaluates our success in attaining the SDG health goal is not much time. Aggressive efforts to improve health worker performance in support of UHC and the quality of care movement is one of the single best approaches to accelerating progress in meeting the SDGs. The key is a multilevel, systems oriented approach that monitors, adapts, and innovates, plus a generous dose of persistence and patience. The continual incremental improvements in the quality of health worker performance will yield the gains in health status necessary to attain the SDG targets.

## Notes

Provenance and peer review: Commissioned; externally peer reviewed.

This article is part of a series based on findings from a global review of Integrated Management of Childhood Illness (IMCI) funded by the Bill and Melinda Gates Foundation. Open access fees were funded by the Health Systems Research Unit, South African Medical Research Council. *The BMJ* peer reviewed, edited, and made the decision to publish the article with no involvement from the Medical Research Council.
